# Effect of contrast material injection duration on arterial enhancement at CT in patients with various cardiac indices: Analysis using computer simulation

**DOI:** 10.1371/journal.pone.0191347

**Published:** 2018-02-23

**Authors:** Toru Higaki, Takeshi Nakaura, Masafumi Kidoh, Hideaki Yuki, Yasuyuki Yamashita, Yuko Nakamura, Fuminari Tatsugami, Yasutaka Baba, Makoto Iida, Kazuo Awai

**Affiliations:** 1 Department of Diagnostic Radiology, Graduate School of Biomedical and Health Sciences, Hiroshima University, Kasumi, Minami-ku, Hiroshima, Japan; 2 Department of Diagnostic Radiology, Faculty of Life Sciences, Kumamoto University, Honjyo, Chuo-ku, Kumamoto, Japan; University of Tennessee Health Science Center, UNITED STATES

## Abstract

Arterial peak enhancement on contrast-enhanced CT (CECT) images is thought to be higher in patients with low cardiac function. Using computer simulations, we tested the hypothesis that the relationship between the cardiac index and the aortic peak CT number (PCTN) is affected by the contrast material (CM) injection duration. We created computer simulation software for the contrast enhancement of various organs and vessels based on the Bae pharmacokinetics model and implemented models for CM transmission within organs and CM diffusion in blood plasma based on the osmotic pressure. Aortic contrast enhancement at coronary- and abdominal CT angiographs (CTA) was simulated for a representative 60-year-old Japanese male 166 cm in height and 65.0 kg in weight. The injection protocol for coronary CTA was: CM dose 45.5 ml, iodine dose, 245 mg/kg body weight (BW); injection duration 8–20 sec in 2-sec increments. For abdominal CTA it was CM dose 74.3 ml; iodine dose 400 mg/kg BW; injection duration 10–40 sec in 5-sec increments. In both protocols the iodine concentration was 350 mgI/ml, osmotic pressure was 590 mOsm/kgH_2_O, and the cardiac index ranged from 0.1–6.0 l/min/m^2^. Under all protocols, the aortic PCTN increased as the injection duration decreased and as the cardiac index rose to the cardiac index value. It then decreased as it exceeded the cardiac index value. At coronary CTA, at an injection duration of 8 or 10 sec, the PCTN exceeded 350 Hounsfield units (HU) at a cardiac index from 0.9–5.6 l/min/m^2^. At an HU value greater than 350, the range of the cardiac index narrowed when the injection duration was 12 sec or longer. On abdominal CTA scans performed with an injection duration of 10-, 15-, or 20 sec, the PCTN exceeded 350 HU at a cardiac index ranging from 0.4–5.3 l/min/m^2^. When the injection duration ranged from 25–40 sec, there was narrowing of the range of the cardiac index at which the PCTN exceeded 350 HU. For coronary and abdominal CTA, contrast enhancement protocols with shorter injection durations yield a diagnostically adequate aortic PCTN at a wide range of cardiac indices.

## Introduction

The primary patient-related factors affecting arterial contrast enhancement at CT are the body size and cardiac output [1–7]. While arterial peak enhancement at contrast-enhanced CT (CECT) is thought to be higher and longer in patients with low cardiac output (CO) [1, 4, 6, 8, 9], controversy surrounds the relationship between arterial peak enhancement and CO [3–5, 9–12]. According to Nakaura et al. [13], when the contrast material (CM) injection duration is short, there is no significant relationship between aortic peak enhancement and CO. Jana et al. [14] reported that aortic enhancement at CECT was decreased in patients with severe cardiac failure due to cardiac arrest or severe cardiogenic shock. Tomizawa et al. [15] observed that the CT number of the ascending aorta on coronary CTA scans exhibited a negative linear correlation with CO, however, the Pearson correlation coefficient between the two parameters was not high (r = -0.44). Thus, the relationship between CO and arterial enhancement at CECT may be more complex than is generally thought. We hypothesized that the relationship between arterial peak enhancement and CO may change with the CM injection duration.

To identify optimal CM injection protocols for coronary- and abdominal CT angiography (CTA), we performed computer simulations to study the relationship between the CO and arterial enhancement under protocols with different injection durations.

## Materials and methods

This retrospective study to validate our computer simulation software was approved by review board of Kumamoto university and Hiroshima University; prior informed patient consent was waived

### Computer simulation software for CECT

**Fig 1:**
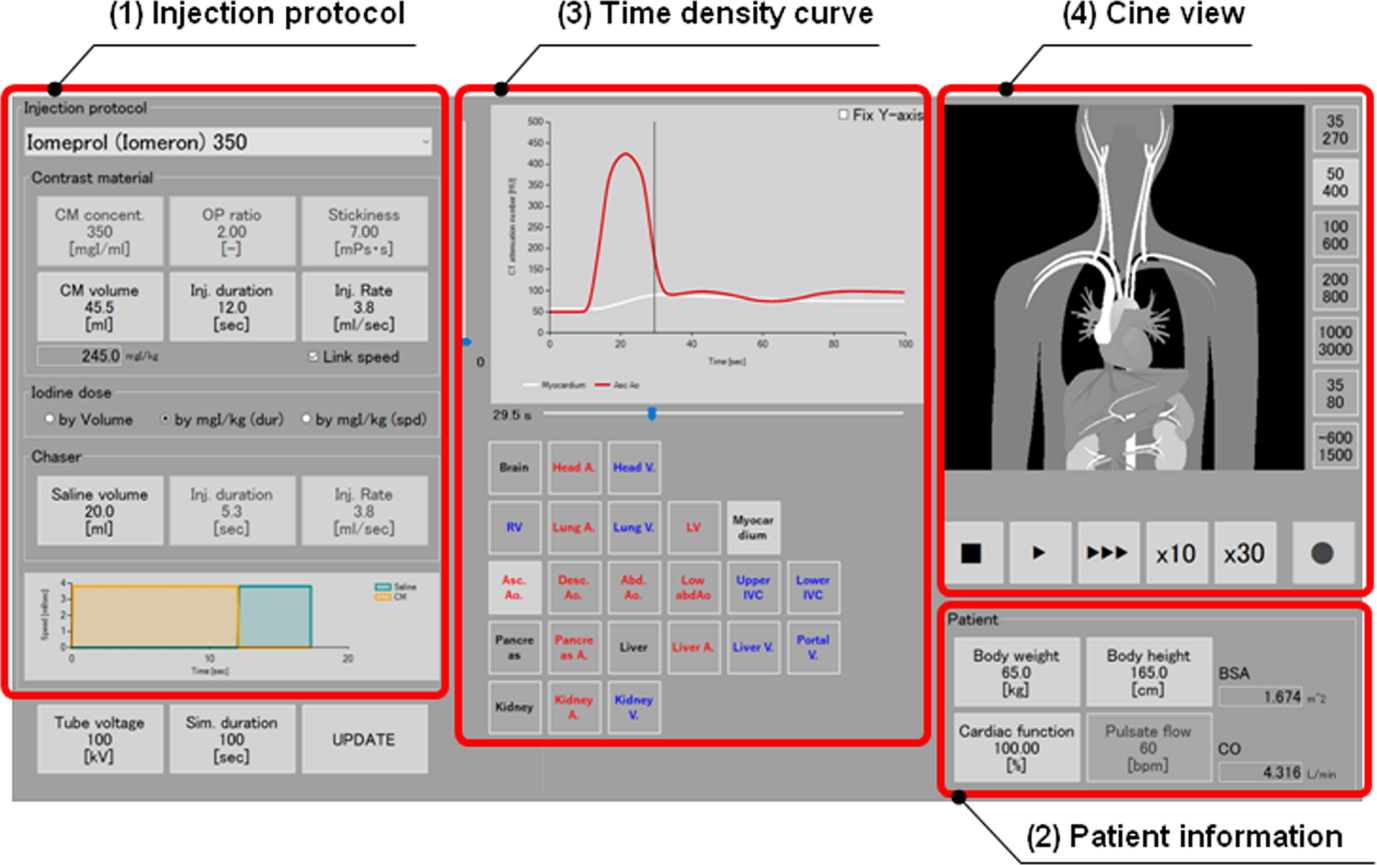
Data input panel of our simulation software. After the input of patient parameters (body weight, height, cardiac output, pulse rate) and the contrast injection protocol [contrast material (CM) volume, injection rate, concentration, osmotic pressure ratio of CM to saline, CM viscosity], our software simulates the time-density curve of selected organs or vessels in patients with an arbitrary constitution for different CM injection protocols.

Our software can simulate the time-density curve of selected organs and vessels in a specific patient with an arbitrary body weight (BW), height, and CO, under protocols that differ with respect to the CM dose, concentration, viscosity, injection rate or duration, and the osmotic pressure ratio of CM to saline. After input of an arbitrary BW, height, and CO on the operator input panel (Fig 1), our software simulates the time-density curve for a specific organ or vessel in a specific patient.

A representative physiology-based pharmacokinetic (PBPK) model for contrast enhancement is that proposed by Bae et al. [1–3]. Refinements of their model have been reported [16, 17]. To develop our simulation software, we modified the original PBPK model by adding organs that were not separately modeled in the PBPK model and by adding the diffusion of CM in the blood and the transmission of CM within an organ or vessel.

#### PBPK model used for our simulations

**Fig 2:**
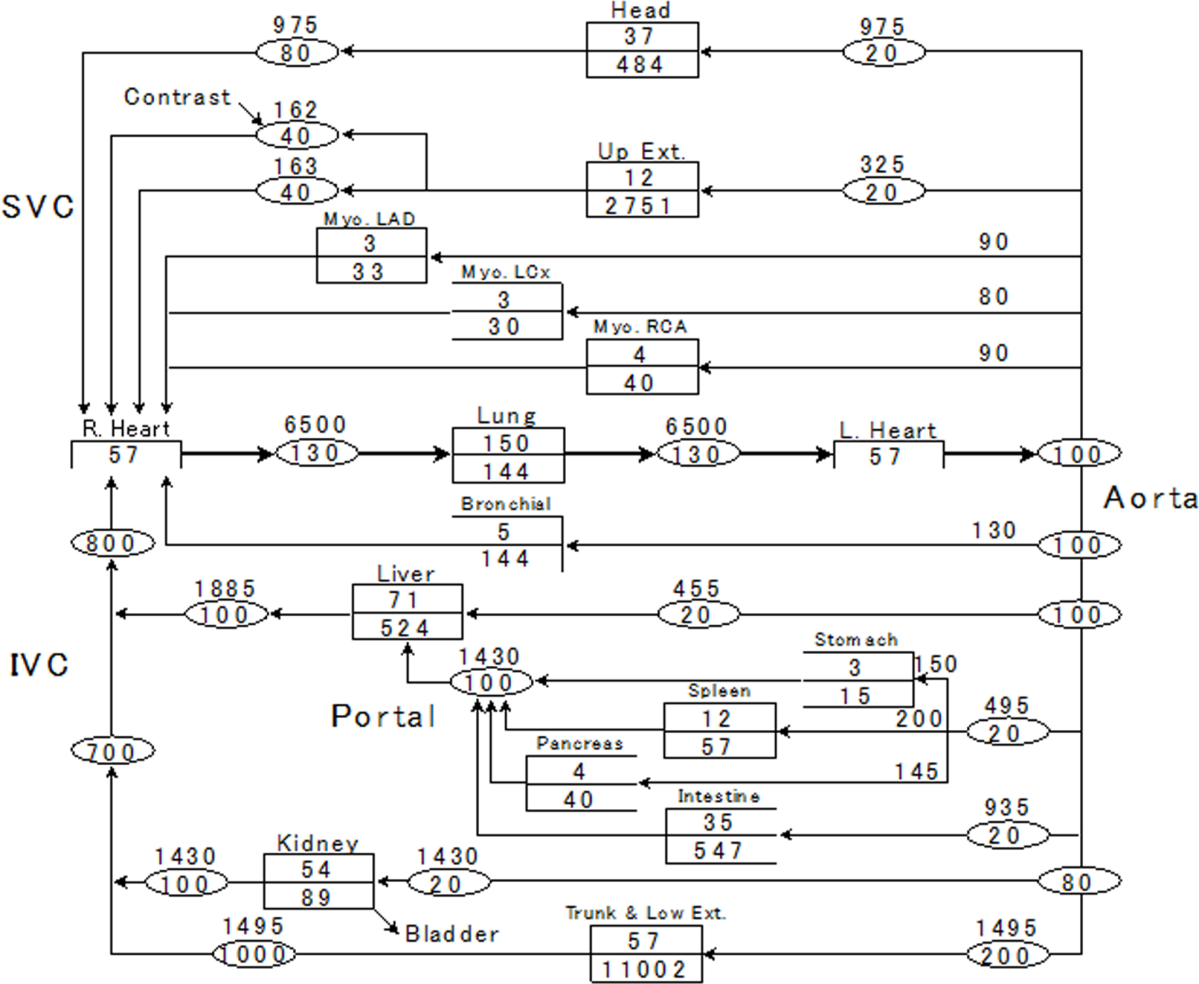
Our pharmacokinetic physiology-based pharmacokinetic model for simulating contrast enhancement in various organs. We modeled the stomach, spleen, pancreas, and myocardium; they are not included in Bae’s model.

We adopted the blood flow rate and the intravascular- and extracellular volume of each compartment used by Bae et al. [3, 8] (Fig 1). In that model, the stomach, spleen, and pancreas were brought together in one compartment, as was myocardial perfusion from the left anterior descending- and the left circumflex branch, and the right coronary artery. We, on the other hand, assigned specific values for the stomach, spleen, pancreas, and myocardium by referring to the blood circulation model proposed by Leggett and Williams [18] (Fig 2). The input and output of each compartment is shown in terms of the CM mass. We applied a blood flow rate and intravascular- and extracellular volume for each organ appropriate for a 60-kg, 1.7-m Japanese male. To simulate a subject with a different BW we adjusted these parameters in direct proportion to the simulated BW.

In our PBPK model, the output flow from the left heart is 6500 ml/min, a volume that corresponds with CO (Fig 2). We calculated an adjustment factor for an arbitrary target CO by dividing the target CO by 6500 ml/min. Then we multiplied the input flow volume of each organ shown in Fig 2 by that adjustment factor and calculated the time-density curve of the arbitrarily-chosen organ or vessel. In the absence of CO data, CO was automatically estimated with the formula [8],

$$CO\left(\frac{ml}{min}\right)=25.3\times H^{-0.725}\times W^{0.425}$$

where H and W are the patient height (cm) and weight (kg), respectively.

#### Simulation of CM diffusion in blood and CM transmission within organs or vessels

**Fig 3:**
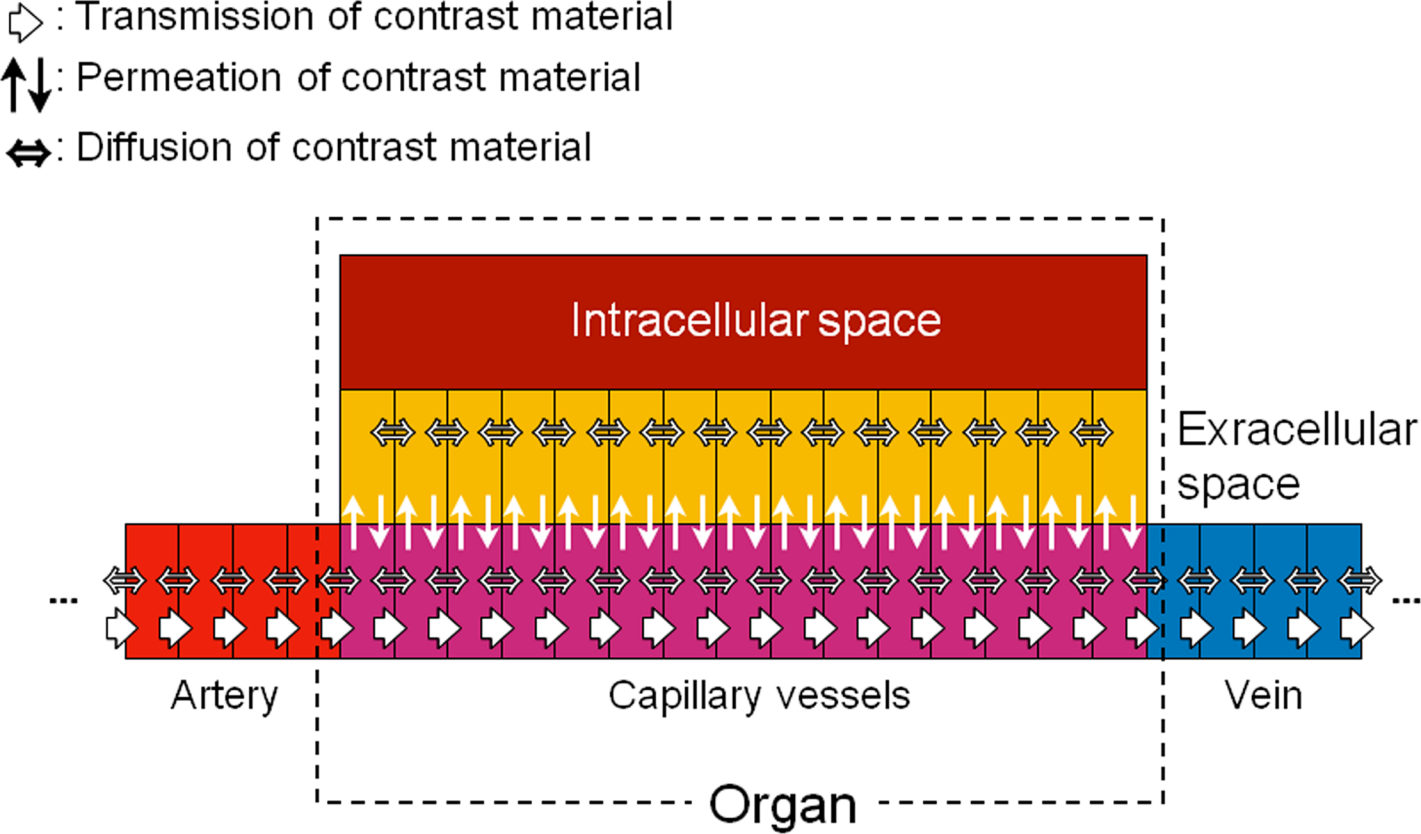
Schema showing a mass transfer between the vascular- and extracellular space in our simulation. Bae’s PBPK model does not model the diffusion of contrast material (CM) in blood or its transmission within one compartment (organ or vessel). Our software includes CM diffusion in blood based on the osmotic pressure. CM diffusion is determined by the osmotic pressure and the CM viscosity. Our software shows transmission within one compartment by dividing the compartment into 15 sub-compartments.

In vivo, 3 mechanisms transport CM; they are CM diffusion in blood, CM transmission by the blood flow, and CM permeation based on the osmotic pressure gradient between the capillary- and extracellular space (Fig 3). As Bae’s PBPK model does not include CM diffusion **i**n the blood and CM transmission within one compartment such as the liver, CM transmission within one compartment was hypothesized to be instantaneous.

Our model for CM diffusion in the blood takes into account the osmotic pressure and CM viscosity. We hypothesized that the CM diffusion speed in blood is in direct proportion to the osmotic pressure of the CM and inversely related to the CM viscosity. To simulate CM transmission within one compartment (one vessel or organ), we subdivided the compartment into some equal sized subcompartments as described later.

#### Calibration of our simulation software

From the 96 below mentioned patients who had undergone test bolus scanning for coronary CTA, we randomly select 30 patients using a random table. Their CECT scans used Iopamidol-370. Because the direct calculation of the absolute value of the diffusion speed of different CMs is difficult, we used Iopamidol-370 (concentration 370 mgI/ml, osmotic pressure ratio to saline 2.76, viscosity 9.1 mPas) as a reference CM. To obtain the diffusion speed, we used data in which the difference between the simulated CM arrival time (CM-AT) and the actual CM-AT and the difference between simulated aortic peak enhancement (PT) and actual PT were minimized.

For CM other than Iopamidol-370, we estimated the diffusion speed by applying the adjustment factor based the ratio of the CM osmotic pressure to saline.

For calibration, we also determined the number of subcompartments in the 30 patients. We divided each compartment into 15 equal units to minimize the difference between the simulated CM-AT and the actual CM-AT and the difference between simulated- and actual PT.

The PCTN was simulated based on the individual patient BW, height, and CO. We used a conversion coefficient between the simulated- and actual PCTN that minimized the mean error between the simulated PCTN and the actual CT number. To investigate the correspondence relation between the CM concentration and the CT number on different scanners we surveyed the relationship between the CM concentration and the CT number on scans acquired on various scanners at all permissible tube voltages.

### Validation study of our computer simulation software

#### Study population

This retrospective study to validate our computer simulation software was approved by our institutional review board; prior informed patient consent was waived. We used existing test bolus injection data from patients who had undergone coronary CTA between August 2014 and June 2015.

The inclusion criteria were *(a)* successful test bolus injection for coronary CTA to evaluate or survey ischemic heart diseases, *(b)* no contraindication for iodinated CM, *(c)* no renal failure (estimated glomerular filtration rate < 45 ml/min/1.73 m^2^), and *(d)* no severe thyroid disease. We used data from 96 patients (52 men, 44 women; median age 72 years, age range 28–88 years; median BW 58.1 kg, weight range 31.9–89.6 kg; median height 158.5 cm, height range 133.3–176.6 cm; median body mass index (BMI) 23.5, BMI range 13.4–32.3; median CO 3.4 l/min, CO range 1.5–6.5 l/min; median cardiac index 2.1 l/min/m^2^, cardiac index range 1.0–3.7 l/min/m^2^). From a random table, we selected 30 of the 96 patients and applied their data to calibrate the simulation software. Data from the other 66 patients were used in the validation study.

CO measurements were with an electrical velocimeter [11] (Aesculon mini, ver. 3.9.6, Osypka Medical GmbH, Berlin, Germany). Based on information provided by the vendor, the measurement error was within 5%.

#### CT scanning and CM injection protocols

All patients were scanned with a 320-detector CT scanner (Aquilion ONE ViSION, Toshiba, Otawawa, Japan). To dilate the coronary arteries, each patient was given 0.3 mg nitroglycerin sublingually 5 min before CT scanning; 33 patients whose heart rate subsequently exceeded 65 beats per minute additionally received landiolol hydrochloride (Corebeta; Ono-Yakuhin-Kogyo, Osaka, Japan).

With a power injector (Dual Shot; Nemoto-Kyorindo, Tokyo, Japan), we delivered 20 ml of CM (iodine concentration 370 mgI/ml; Iopamiron 370; Bayer Yakuhin, Osaka, Japan) via a 20-gauge catheter into the antecubital vein. The injection duration and injection rate were 5 sec and 4.0 ml/sec, respectively. Flushing was with 20 ml of physiological saline delivered at the same injection rate.

To monitor the ascending aorta we obtained dynamic low-dose (120 kVp, 40 mAs) scans; the detector collimation was 1 x 4 mm and the interscan interval was 1.0 sec. Acquisition of the dynamic monitoring scans began 10 sec after the start of CM injection. Image reconstruction was in a 32-cm display field of view depending on the patient physique.

#### Analysis

**Fig 4:**
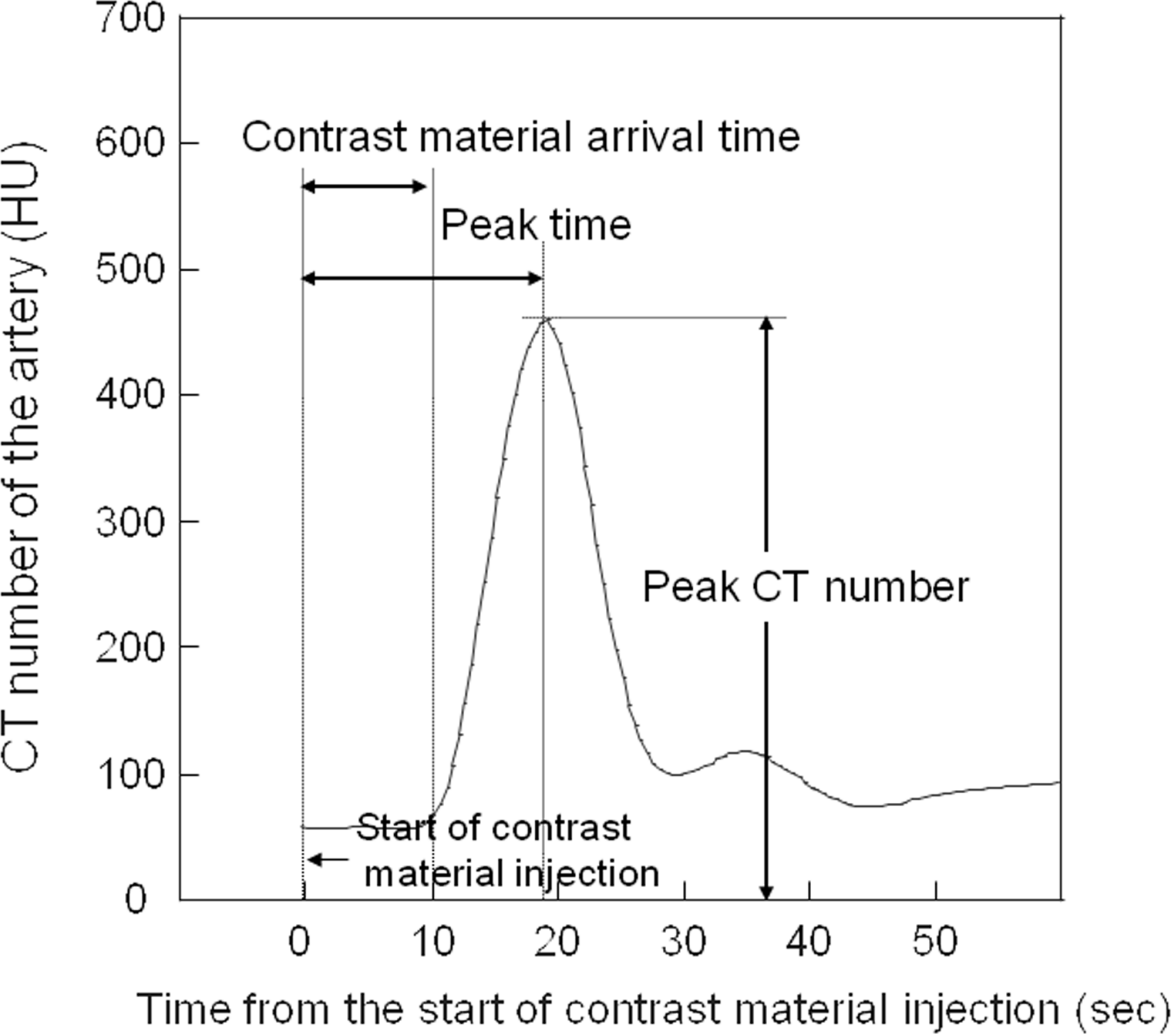
Time curve of arterial enhancement of the aorta. We defined the arrival time (AT) of the contrast material as the point 2 sec before the attenuation value was 10 HU greater than on unenhanced CT scans. The peak time (PT) was defined as the time between the start of contrast material injection and the time at which the arterial CT number peaked.

We set a region of interest in the ascending aorta to obtain a time-density curve for each patient. CM-AT, PT, and PCTN were obtained on time-density curves by connecting all time points for each patient. We defined AT as the point 2 sec before the aortic attenuation value on the time-density curve was 30 Hounsfield units (HU) greater than on unenhanced CT scans (Fig 4).

To validate the clinical applicability of our simulation software we compared the actual and estimated CM-AT, the actual and estimated PT, and the actual and estimated PCTN. For comparisons we grouped patients by their cardiac index [(cardiac index) ≤ 1.5, 1.5 < (cardiac index) ≤ 2.0, 2.0 < (cardiac index) ≤ 2.5, and 2.5 < (cardiac index)].

### Simulation of arterial enhancement in patients with various COs

We simulated the relationship between the cardiac index and CM-AT, the cardiac index and PT, and the cardiac index and PCTN at different injection durations. The simulated cardiac index range was 0.1–6.0 l/min/m^2^.

On an annual basis, most CT studies in Japan are performed in patients in their 60’s and 70’s [19]. Therefore, in our simulations, we used data representative of a 60-year-old Japanese male [20]. The CM injection protocol for the enhancement of the ascending aorta on coronary CTA scans was: CM dose 45.5 ml, iodine concentration 350 mg/ml, iodine dose 245 mg/kg BW, injection duration from 8–20 sec in 2-sec increments. To simulate enhancement of the abdominal aorta on abdominal CTA scans the CM dose was 74.3 ml, iodine concentration 350 mg/ml, iodine dose 400 mg/kg BW, injection duration from 10–35 sec in 5-sec increments. We adopted uniphasic CM injection protocols in all simulation. Therefore, the injection rate was constant during the injection duration in all simulated protocols.

A CT number of 350 HU was set as the threshold for adequate aortic enhancement [4, 21, 22].

### Statistical analysis

To validate our simulation software, the relationship between the actual- and estimated CM-AT, the actual and estimated PT, and the actual and estimated PCTN was calculated, as were the intraclass correlation coefficients. We also calculated the 95% confidence interval (CI) of the population mean for the actual CM-AT, PT, and PCTN, and compared the mean of the estimated CM-AT, PT, and PCTN for each cardiac index level.

## Results

### Validation study

**Fig 5:**
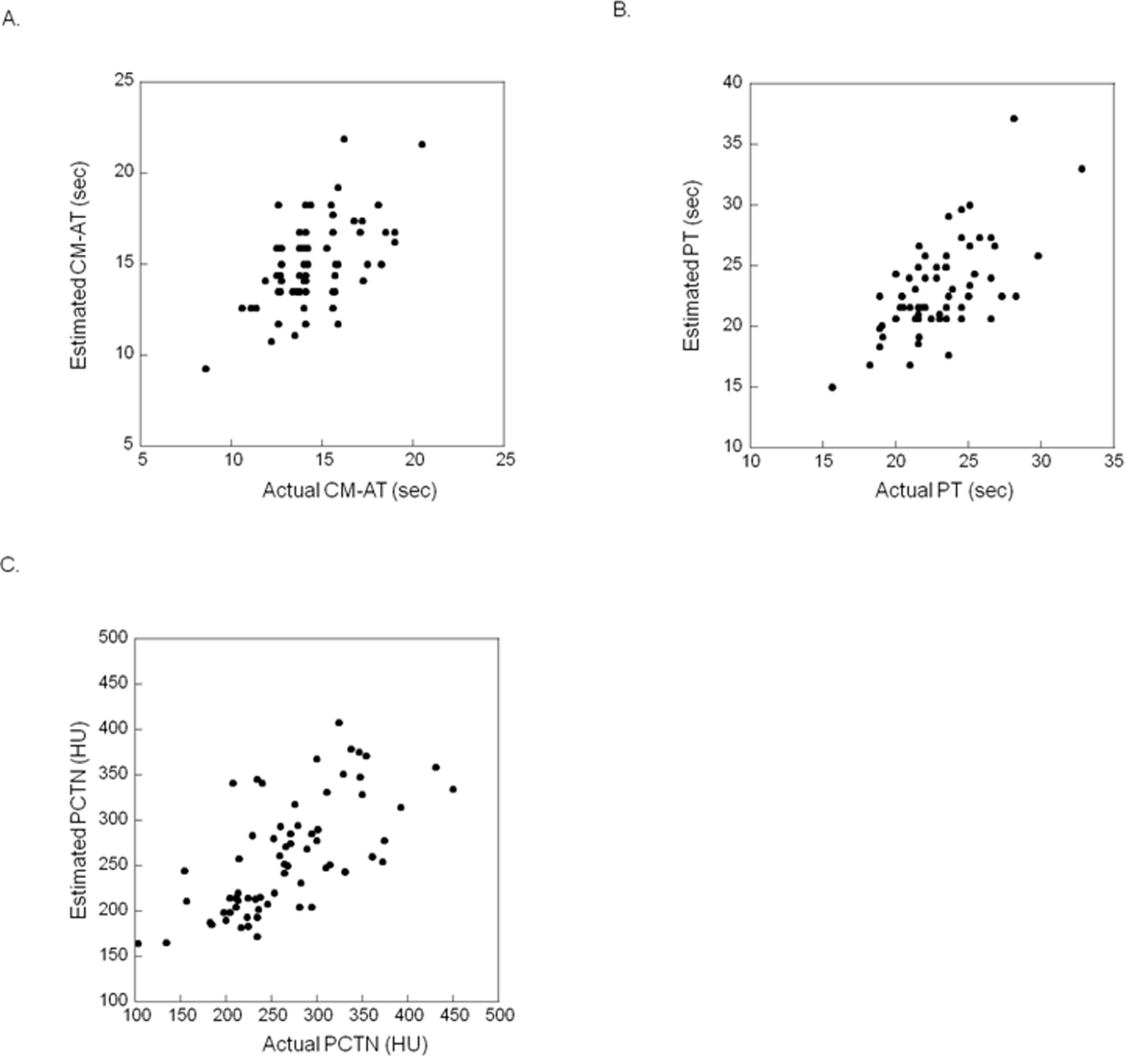
**A. Relationship between the actual and the estimated contrast material arrival time (CM-AT).** The intraclass correlation coefficient between the actual and the estimated AT was 0.53 (95% CI). **B. Relationship between the actual and the estimated contrast material peak time (PT).** The intraclass correlation coefficient between the actual and the estimated PT was 0.63 (95% CI: 0.45–0.75). **C. Relationship between the actual and the estimated peak CT number of the aorta (PCTN).** The intraclass correlation coefficient between the actual and the estimated PCTN was 0.67 (95% CI: 0.51–0.78).

The relationship between the actual and estimated CM-AT, PT, and PCTN is shown in Fig 5A, 5B, and 5C. The intraclass correlation coefficient (95% CI) between the actual and the estimated CM-AT, PT, and PCTN was 0.53 (0.34–0.69), 0.63 (0.45–0.75), and 0.67 (0.51–0.78), respectively.

Except for the cardiac index of 1.6–2.0 l/min/m^2^, the mean of the estimated CM-AT and of the estimated PT was within the 95% CI of the population mean of the actual values (Tables 1 and 2). As shown in Table 3, the mean of the estimated PCTN was also within the 95% CI of the population mean of the actual PCTN at all cardiac index levels.

**Table 1:** Mean of the estimated CM-AT and 95% CI of the population mean of the actual CM-AT.

Cardiac Index (l/min/m^2^)	Mean of the estimated CM-AT (sec)	95% CI of the population mean of the actual CM-AT (sec)
-1.5	20.3	12.4–21.0
1.6–2.0	16.9	14.5–16.6
2.1–2.5	14.5	13.7–14.9
2.6-	11.6	11.0–14.5

Abbreviations:

CM-AT, contrast material arrival time at the ascending aorta

CI, confidence interval

**Table 2:** Mean of the estimated PT and 95% CI of the population mean of the actual PT.

Cardiac Index (l/min/m^2^)	Mean of the estimated PT (sec)	95% CI of the population mean of the actual PT (sec)
-1.5	32.5	21.6–33.7
1.6–2.0	25.9	22.7–25.3
2.1–2.5	21.9	21.9–23.4
2.6-	18.0	18.0–21.6

Abbreviations:

PT, peak time of the contrast material in the ascending aorta

CI, confidence interval

**Table 3:** Mean of the estimated PCTN and 95% CI of the population mean of the actual PCTN.

Cardiac Index (l/min/m^2^)	Mean of the estimated PCTN (HU)	95% CI of the population mean of the actual PCTN (HU)
-1.5	213	85–342
1.6–2.0	243	225–303
2.1–2.5	253	241–284
2.6-	340	272–345

Abbreviations:

PCTN, peak CT number of the contrast material in the ascending aorta

CI, confidence interval

### Simulation of arterial enhancement in patients with various COs

**Fig 6:**
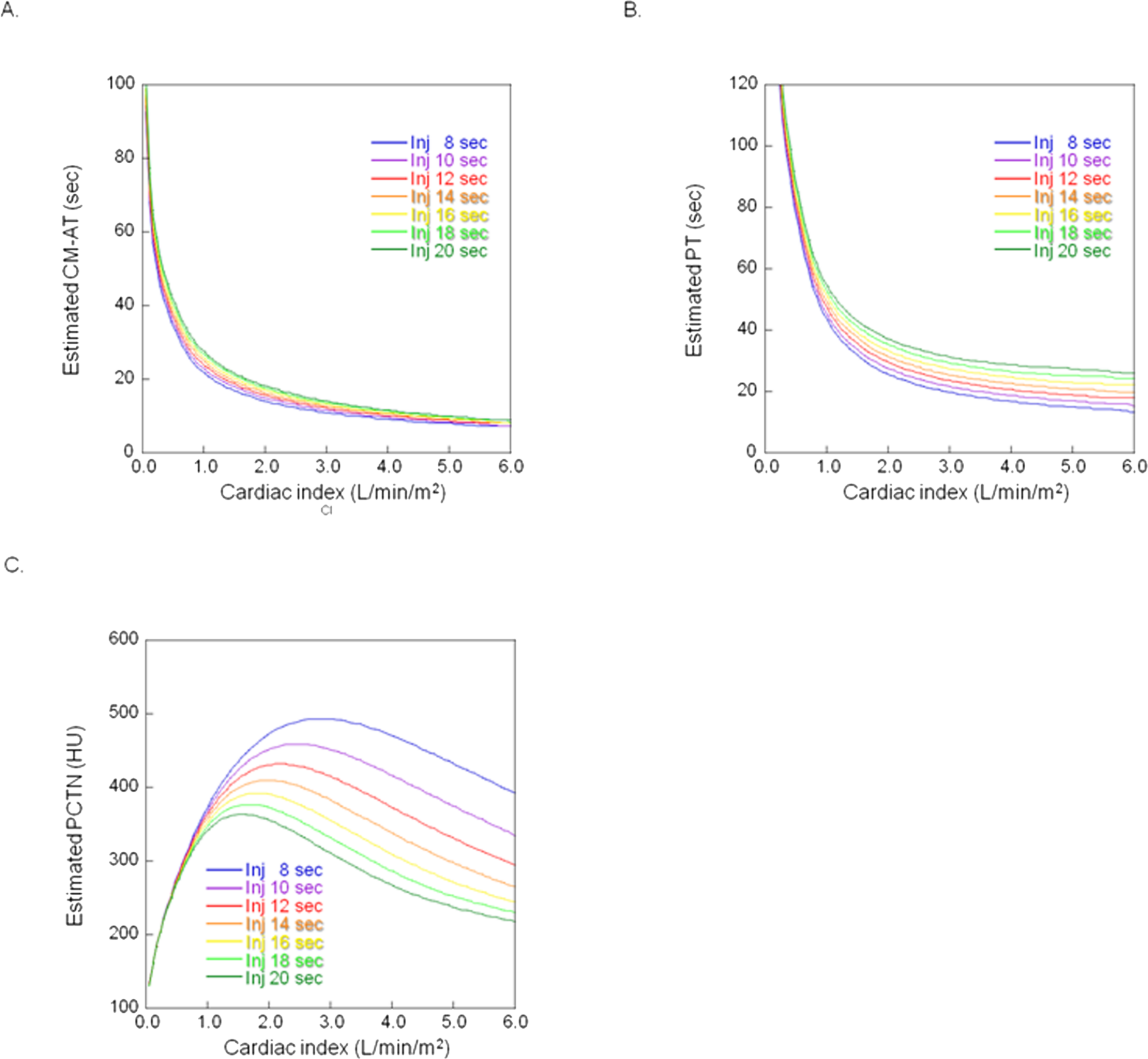
**A. Relationship between the cardiac index and the estimated contrast material arrival time (CM-AT) at coronary CT angiography.** Inj: injection duration. **B. Relationship between the cardiac index and the estimated peak time of the contrast material (PT) at coronary CT angiography.** Inj: Injection duration. **C. Relationship between the cardiac index and the estimated peak CT number (PCTN) of the thoracic ascending aorta at coronary CT angiography.** Inj: Injection duration.

**Fig 7:**
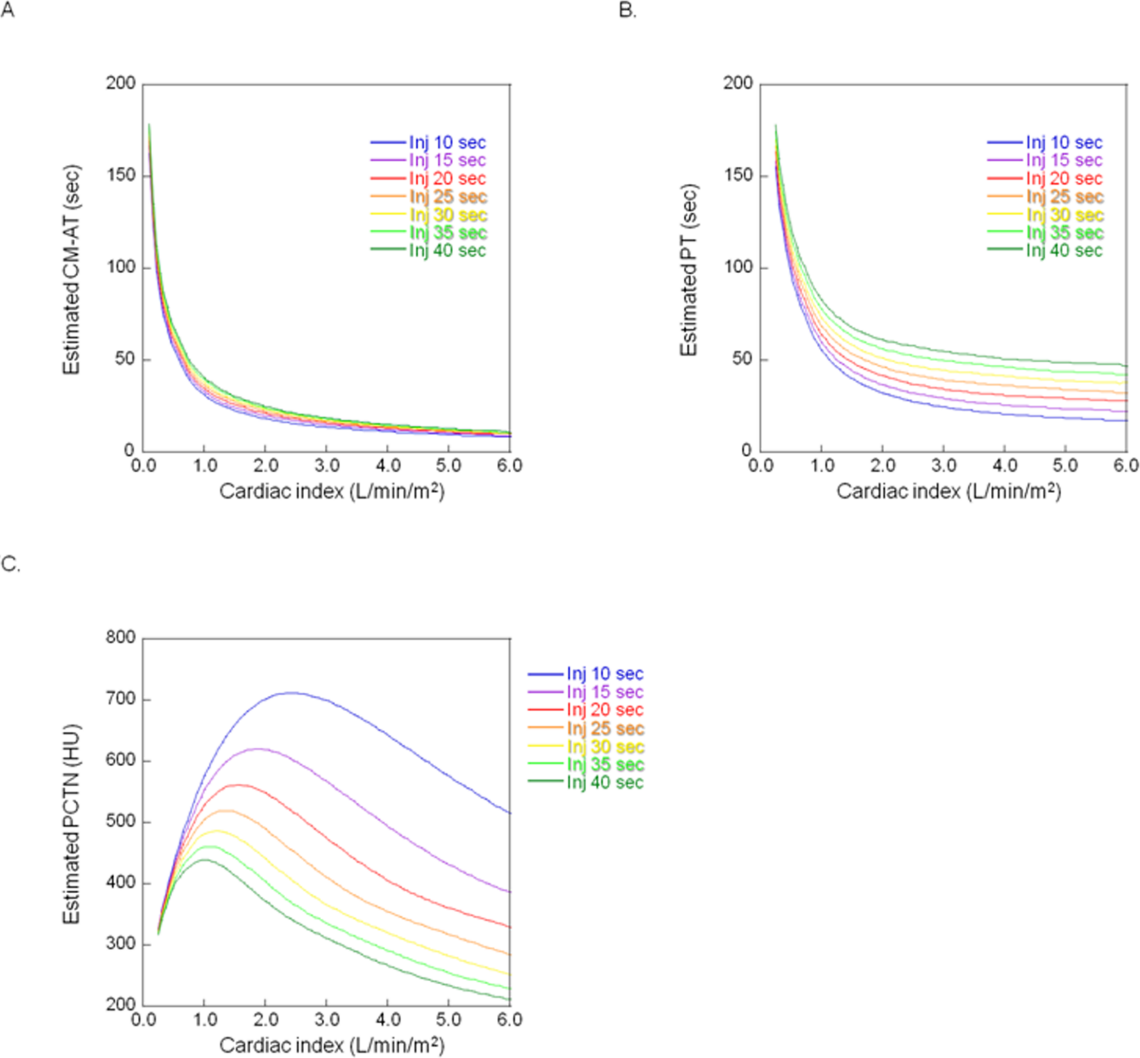
**A. Relationship between the cardiac index and the estimated contrast material arrival time (CM-AT) of at abdominal CT angiography.** Inj: Injection duration. **B. Relationship between the cardiac index and the estimated peak time (PT) of contrast material at abdominal CT angiography.** Inj: Injection duration. **C. Relationship between the cardiac index and the estimated peak CT number (PCTN) of the abdominal aorta at abdominal CT angiography.** Inj: Injection duration.

On coronary and abdominal CTA scans, the estimated CM-AT and the estimated PT decreased exponentially as the cardiac index increased (Figs 6A, 6B, 7A and 7B).

Under the protocols for coronary and abdominal CTA, the PCTN increased as the cardiac index rose to the cardiac index value. It then decreased as the cardiac index increased until it exceeded the cardiac index value (Figs 6C and 7C). For example, under the coronary CTA protocol using an 8-sec injection time, the PCTN increased with CO when the cardiac index was below 2.8 l/min/m^2^; it decreased when it exceeded 2.8 l/min/m^2^ (Fig 6C).

On simulated coronary CTA scans obtained with an injection duration of 8 or 10 sec, the PCTN was larger than 350 HU at the cardiac index ranging from 0.9–5.6 l/min/m^2^ (Table 4). When the injection duration was 12 sec or longer, the cardiac index at which the PCTN exceeded 350 HU narrowed (Table 5).

**Table 4:** Cardiac index at which the PCTN of the ascending aorta exceeded 350 HU.

	Cardiac index (l/min)	
Injection duration (sec)	Minimum	Maximum
8	0.85	> 6.00
10	0.90	5.60
12	0.90	4.50
14	0.90	3.75
16	0.95	3.10
18	1.00	2.60
20	1.05	2.15

Abbreviations:

PCTN, peak CT number

**Table 5:** Cardiac index at which the PCTN of the abdominal aorta exceeded 350 HU.

	Cardiac index (l/min)	
Injection duration (sec)	Minimum	Maximum
10	0.30	> 6.00
15	0.30	> 6.00
20	0.35	5.30
25	0.35	4.10
30	0.35	3.25
35	0.35	2.70
40	0.35	2.25

Abbreviations:

PCTN, peak CT number

In abdominal CTA simulations, at injection durations of 10-, 15-, or 20 sec, the PCTN exceeded 350 HU at the cardiac index from 0.4–5.3 l/min/m^2^ (Table 5). However, when the injection duration was 25–40 sec, the CO range in which the PCTN was more than 350 HU narrowed (Table 5).

## Discussion

As the mean of the estimated PCTN was within the 95% CI of the population mean of the actual PCTN at all cardiac index levels, we concluded that our simulation software accurately predicted the PCTN. On the other hand, the mean of the estimated CM-AT and PT was out of the 95% CI range of the population mean of the actual CT-AT and PT in patients with a cardiac index of 1.6–2.0 l/min/m^2^. Therefore, our simulation was less accurate for CM-AT and PT than for PCTN. Although we cannot explain this result, we think that there might be additional factors that play a role in the body size index and CO, e.g. vascular resistance and CM retention in the upper arm veins, that were not included in our model. Therefore, actual measurement of the CM-AT using a test bolus injection or the bolus tracking technique may be necessary for the accurate prediction of the CM-AT and PT.

Based on their computer simulation- and porcine studies, Bae et al. [3] reported that a CO reduction resulted in slower CM clearance and higher, prolonged CM enhancement. At a given injection duration, we found that on coronary and abdominal CTA scans, the PCTN increased with the cardiac index until it reached the cardiac index value; it subsequently decreased as the cardiac index increased. When the cardiac index exceeds a specific value, CM may be washed out by the faster blood flow, resulting in a lower arterial PCTN. On the other hand, when the cardiac index is lower than a specific value, a lower CO may elicit CM stasis, thereby expediting CM dilution in the blood due to high CM osmolality.

When the injection duration was 8–10 sec in our coronary CTA protocols, the PCTN was higher than 350 HU in a wide cardiac index range (0.9–5.6 l/min/m^2^). However, under protocols with longer, i.e. 18- and 20-sec injection durations, a PCTN exceeding 350 HU was observed in limited cardiac index ranges (1.0–2.6 and 1.1–2.2 l/min/m^2^). Consequently, sufficient arterial enhancement can be obtained only in patients with lower cardiac function. On the other hand, with shorter injection-duration protocols, enhancement may be sufficient in patients with various degrees of cardiac function. This may also be true with respect to abdominal CTA; in our study, injection durations of 20 sec or less yielded sufficient arterial enhancement. Thus, for abdominal CTA, CM injection protocols with shorter injection durations may be robust, independent of cardiac function.

Although we calibrated our software not only for PTCN but also for CM-AT and PT, the simulated AT and PT values showed no strong correlation with the actual measured values. We think that one reason for the difficulty of estimating AT and PT with our software is related to factors that were not modeled in our simulation (vascular resistance and collateral circulation). Investigations are underway to address this issue.

To perform simulations reflecting the pharmacodynamics of CM, we examined CM transmission within an organ and CM diffusion attributable to the CM osmolarity. The diffusion model may make it possible to simulate the arterial PCTN at a lower cardiac index.

Our study has some limitations. First, we measured cardiac function with an electrical velocimeter rather than a direct measurement method such as the pulmonary artery thermodilution technique that employs a Swan-Gantz catheter [23]. However, as the measurement error of the velocimeter is within 5%, we think its use is acceptable. Second, the median CO in our representative Japanese male was 3.4 l/min (range 1.5–6.0 l/min), this value might be lower than in Western populations where the CO range for a normal subject is 4.0–8.0 l/min [24]. At present, our findings cannot be extrapolated to Western populations. Third, although the number of our patients with a CO below 2.0 l/sec (n = 23) may be considered to be low, as patients with significant cardiac disease may be at increased risk for reactions to CM [25], the enrollment of high numbers of patients with low CO is difficult.

In conclusion, our study showed that at simulated coronary and abdominal CTA, aortic PCTN increased with the cardiac index until it reached a specific value. It subsequently decreased as the index increased beyond a specific value. We also documented that contrast enhancement protocols with shorter injection durations yield diagnostically sufficient arterial peak enhancement at a wide range of cardiac indices.

## Supporting information

S1 Table Raw Data used for calibration and validation of our simulation software. (XLSX)
